# Plasma coenzyme Q_10_ status is impaired in selected genetic conditions

**DOI:** 10.1038/s41598-018-37542-2

**Published:** 2019-01-28

**Authors:** Raquel Montero, Delia Yubero, Maria C. Salgado, María Julieta González, Jaume Campistol, Maria del Mar O’Callaghan, Mercè Pineda, Verónica Delgadillo, Joan Maynou, Guerau Fernandez, Julio Montoya, Eduardo Ruiz-Pesini, Silvia Meavilla, Viruna Neergheen, Angels García-Cazorla, Placido Navas, Iain Hargreaves, Rafael Artuch

**Affiliations:** 1 Inborn errors of metabolism Unit, Institut de Recerca Sant Joan de Déu, Barcelona, Spain; 2 CIBER de Enfermedades Raras (CIBERER), Instituto de Salud Carlos III Spain, Madrid, Spain; 3 Department of Genetic and Molecular Medicine, Institut de Recerca Sant Joan de Déu, Barcelona, Spain; 4 Departamento de Bioquimica y Biologia Molecular y Celular, Universidad Zaragoza-Instituto de Investigación Sanitaria de Aragón (IISAragon), Zaragoza, Spain; 5 Neurometabolic Unit, National Hospital, Queen Square, London, UK; 6 Centro Andaluz de Biología del Desarrollo, Universidad Pablo de Olavide, Sevilla, Spain; 7 School of Pharmacy, Liverpool John Moores University, Liverpool, UK

## Abstract

Identifying diseases displaying chronic low plasma Coenzyme Q_10_ (CoQ) values may be important to prevent possible cardiovascular dysfunction. The aim of this study was to retrospectively evaluate plasma CoQ concentrations in a large cohort of pediatric and young adult patients. We evaluated plasma CoQ values in 597 individuals (age range 1 month to 43 years, average 11 years), studied during the period 2005–2016. Patients were classified into 6 different groups: control group of healthy participants, phenylketonuric patients (PKU), patients with mucopolysaccharidoses (MPS), patients with other inborn errors of metabolism (IEM), patients with neurogenetic diseases, and individuals with neurological diseases with no genetic diagnosis. Plasma total CoQ was measured by reverse-phase high-performance liquid chromatography with electrochemical detection and ultraviolet detection at 275 nm. ANOVA with Bonferroni correction showed that plasma CoQ values were significantly lower in the PKU and MPS groups than in controls and neurological patients. The IEM group showed intermediate values that were not significantly different from those of the controls. In PKU patients, the Chi-Square test showed a significant association between having low plasma CoQ values and being classic PKU patients. The percentage of neurogenetic and other neurological patients with low CoQ values was low (below 8%). In conclusión, plasma CoQ monitoring in selected groups of patients with different IEM (especially in PKU and MPS patients, but also in IEM under protein-restricted diets) seems advisable to prevent the possibility of a chronic blood CoQ suboptimal status in such groups of patients.

## Introduction

Coenzyme Q_10_ (CoQ) is a lipid that acts in the mitochondrial respiratory chain as an electron transporter essential for ATP synthesis and serves as a lipophilic antioxidant, among other functions^1^. The benzoquinone ring of CoQ is derived from tyrosine, while the polyprenyl side-chain comes from acetyl-CoA, through the mevalonate pathway, which is common to the synthesis of other lipids such as dolichol and cholesterol, in a tightly regulated process^2^. Blood CoQ status depends on liver biosynthesis and is also the result of dietary sources that can influence plasma CoQ concentrations, contributing up to 25% of the total amount^3^. For CoQ, all tissues and cells are able to synthesize the sufficient amounts necessary for their different biological functions; therefore, no noticeable degree of uptake of CoQ seems to occur between the blood and tissues^4^.

In blood, CoQ is transported by the lipoprotein cholesterol (Chol) transporters^5^. CoQ has been shown to be very efficient in preventing low-density lipoprotein (LDL) oxidation^6^. Since oxidized LDL is considered to have a key function in the development of the atherosclerotic process leading to cardiovascular diseases, treatment with CoQ to prevent this oxidation may have therapeutic value. Accordingly, clinical trials of CoQ supplementation to patients suffering from cardiovascular diseases have reported a reduction in the level of biochemical markers associated with pathology and major adverse cardiovascular events^7,8^. Therefore, identifying diseases displaying chronic low plasma CoQ values may be important to prevent possible cardiovascular dysfunction. The relationship between total blood Chol and CoQ is based on the rationale that both of these molecules share a common biosynthetic pathway, the mevalonate pathway^9^. Thus, the simultaneous measurement of plasma CoQ and Chol levels is of interest to assess the relationship between the presence of these two lipids in the blood^10^ and may therefore predict the potential for the oxidative damage of the cholesterol transporter lipoproteins^11^.

Some genetic and environmental conditions have been associated with a decreased level of plasma CoQ values in pediatric patients^12–18^. Thus, plasma CoQ status may be a valuable biomarker for certain diseases, both for diagnosis and treatment monitoring. Moreover, the key role of CoQ in the protection of Chol lipoprotein against free-radical damage strongly advocates the identification of patients presenting with chronic low plasma CoQ values.

In view of the diagnostic potential of plasma CoQ status, the aim of this study was to retrospectively evaluate plasma CoQ concentrations and CoQ/Chol ratios in a cohort of pediatric and young adult patients from a period of 12 years. We focused on those patients presenting with low plasma CoQ values, assessing genetic and environmental factors that could influence plasma CoQ status.

## Methods

### Subjects

We retrospectively evaluated our database containing 597 individuals (age range 1 month to 43 years, average 11 years), studied during the period 2005–2016 in Hospital Sant Joan de Déu (Barcelona) and in the Great Ormond Street Hospital (London). Patients were classified into 6 different groups: control group of healthy participants, phenylketonuric (PKU) patients due to mutations at the *PAH* gene encoding phenylalanine hydroxylase, patients with mucopolysaccharidoses (MPS), patients with other inborn errors of metabolism (IEM), patients with neurogenetic diseases, and lastly individuals with neurological diseases with no genetic diagnosis. Details of these cohorts of patients are provided in Table 1 and in the Supplementary Material 1. From the latter group, we selected 9 cases who were found to have low plasma CoQ values associated with a neurological syndrome for genetic diagnosis through next-generation sequencing (NGS). Total Chol values were also analyzed from the PKU, MPS, and IEM groups (Table 1). The criteria for the group classification were: i) Patients under restricted dietary treatment and at the risk of a suboptimal CoQ status: PKU and IEM patients. ii) Patients with MPS, since these patients may present with low CoQ values, but they are not under dietary restriction. iii) Patients with neurogenetic conditions just to identify diseases that may present with low plasma CoQ levels. iv) Neuropediatric patients with no diagnosis, to assess whether CoQ status would be a surrogate biomarker for the diagnosis of genetic related primary or secondary CoQ deficiencies.

**Table 1:** Plasma CoQ and Chol concentrations in controls and 5 groups of patients.

Subject groups: age range (average)	Plasma CoQ (µmol/L)	Plasma Chol (mmol/L)	Plasma CoQ/Chol (µmol/mol)	Number of low CoQ values cases
**PKU (n = 113)** 9m-43y (16.8)	0.20–1–18 (0.49) SD = 0.20	2.46–8.36 (3.74) SD = 0–74	51–324 (132) SD = 49.7	CoQ: 37/113 CoQ/Chol: 33/113
**MPS (n = 44)** 3–25 y (11.4)	0.16–0.93 (0.40) SD = 0.17	3.14–6.35 (4.40) SD = 0.85	29–201 (94) SD = 40.3	CoQ 24/44 CoQ/Chol 17/30
**IEM (n = 61)** 6m-40y (10.4)	0.18–1.21 (0.55) SD = 0.19	2.27–5.89 (4.04) SD = 0.85	69–291 (139) SD = 46.2	CoQ 11/61 CoQ/Chol 13/61
**Neurogenetic conditions (n = 99)** 1m-27y (10.3)	0.25–1–30 (0.67) SD = 0.21	n.a	n.a	CoQ 5/99
**Other neurological disorders (n = 197)** 1m-35y (8.2)	0.20–1.67 (0.68) SD = 0.27	n.a	n.a	CoQ 19/197
**Control group (n = 83)** 8m-22y (10.6)	0.38–1.34 (0.65) SD = 0.24	2.46–5.88 (4.01) SD = 0.77	101–283 (163) SD = 51	

Reference values are stated as range (defined as 2.5 and 97.5 percentiles), average (in brackets) and SD. For patient groups, data are represented as range (average and standard deviation). Age is expressed as range (average). Only the PKU group showed a significantly higher average age when compared with the other groups. In PKU cases elder than 22 years of age, the % of low plasma CoQ values was slightly higher when compared with to those younger than 22 years of age (8 out of 22). *m: months of age. y: years of age.

Controls were healthy children with no chronic pharmacological treatments submitted to our Hospital for minor surgical interventions (mainly phimosis, adenoids and tympanic drainage). The different groups of diseases studied here were excluded based on: (1) biochemical data: most of them underwent the expanded newborn screening programs that include PKU and other IEM groups. (2) Clinical data: controls were healthy children with no neurological complications, while the MPS, neurogenetic and other neuropediatric patients included in the present study were severely handicapped.

From the 113 PKU patients, the type of PAH gene mutation was determined in 89 patients, as reported^19^ (Supplementary Data Set). The assigned value study classifies the mutations as 4 categories (1 classic PKU, 2 moderate PKU, 4 Mild PKU, and 8 mild HPA) for every mutant *PAH* allele. Thus, the sum of the scores obtained for every allele led to the final classification of patients. In 65 out of 113 PKU patients, the predicted residual PAH activity was also calculated as previously reported^20^. Both variables (assigned value and residual PAH activity) were studied as predictors of the risk of developing low plasma CoQ values. All PKU patients were under dietary treatment at the time of the study, as previously reported^21^.

For the 44 MPS patients (Table 1), we increased the number of patients studied in our previous work^15^ from Sanfilipo to include patients with other MPS disorders (Supplementary Material 1). For the 61 IEM patients, we compared CoQ values in a subgroup on a carbohydrate-restricted diet (galactosemic and fructosemia) with those of a group on protein-restricted diets (homocystinurias, organic acidurias, urea cycle defects, and other aminoacidopathies) (Supplementary Material 1).

Exclusion criteria included patients taking CoQ. Blood samples were taken in the morning after an 8–12 h fasting period. Blood samples were collected into evacuated glass tubes containing heparin. Blood was immediately centrifuged at 4 °C (1500xg), and the plasma samples were stored at −80 °C until analysis.

### Ethical issues

All patient samples were obtained in accordance with the 2013 revised Helsinki Declaration of 1964. For biochemical and genetic investigations, informed consent was collected from patients or their guardians. The Ethical Committee of Sant Joan de Déu Hospital approved the study.

### Biochemical methods

**Figure 1:**
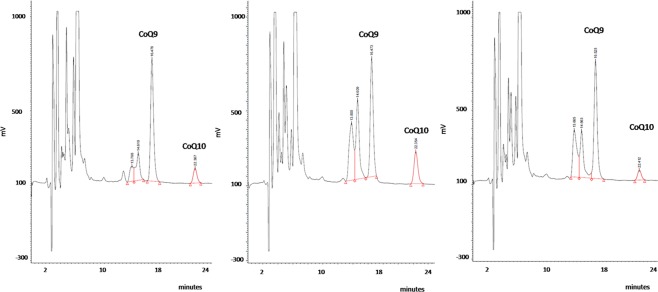
Typical chromatograms (HPLC with electrochemical detection) from: Left panel. Commercial control (Coenzyme Q_10_ Chromsystens, level 1 (Ref. 0092): CoQ_10_ = 0.56 µmol/L), based on serum matrix. Middle panel. Human plasma sample with CoQ_10_ = 1.18 µmol/L. Right panel. Human plasma sample with CoQ_10_ = 0.38 µmol/L. Samples, calibrators and controls are spiked with internal standard (Coenzyme Q_9_ (CoQ_9_)).

Serum total Chol values were analyzed by the automated cholesterol oxidase procedure in an Architect autoanalyzer (Abbot). Plasma total CoQ, the sum of the reduced form ubiquinol plus the oxidized ubiquinone, was measured by reverse-phase high-performance liquid chromatography with electrochemical detection and with ultraviolet detection at 275 nm, as previously reported^22,23^. Plasma CoQ determination was accredited by the norm ISO15189 (ENAC). Reports regarding this accreditation are available upon request. In Fig. 1, typical chtomatograms from internal quality control material and human plasma samples are depicted. As we can see in the Fig. 1, the separation of CoQ and internal standard is optimal and both electrochemical and ultraviolet detection systems are specific for CoQ analysis in complex matrixes such as plasma, HPLC-electrochemical detection having a greater sensitivity when compared with ultraviolet detection or other approaches.

### Genetic analysis

A PKU and MPS mutation study was performed by Sanger sequencing and NGS technology, as previously reported^24^. For the 9 selected patients with unknown neurological conditions and plasma CoQ deficiency, we applied a commercial panel (TruSight One Sequencing Panel, Illumina) and a NextSeq. 500 sequencer (Illumina) to screen a maximum number of candidate genes.

### Statistical methods

The Pearson correlation test was used to determine the correlations among plasma CoQ and Chol values and the patient age and to correlate PAH residual activity with plasma CoQ concentration. The ANOVA with Bonferroni correction test was applied to compare plasma CoQ, Chol, and CoQ/Chol values among the different cohorts of patients and controls. Student T test was used to compare CoQ status in IEM patients with different dietary treatment (carbohydrate and protein-restricted). Chi-Square test was used to search for an association between the type of mutations (classified according to 25) and the CoQ status (deficient or not) in the PKU cohort. Statistical calculations were performed using SPSS 23.0 software.

## Results

In the control group, a highly positive correlation was observed between plasma CoQ and total Chol values (Pearson test; r = 0.523; p < 0.0001) (Supplementary Figure 1). No correlation was observed between the age and plasma CoQ and CoQ/Chol values in controls under 22 years of age. Thus, a unique reference interval was established for this group (Table 1). We defined low plasma CoQ values as those below the lowest limit of the reference interval established in our laboratory, which was 0.38 µmol/L corresponding to the 2.5 percentile (Table 1).

**Figure 2:**
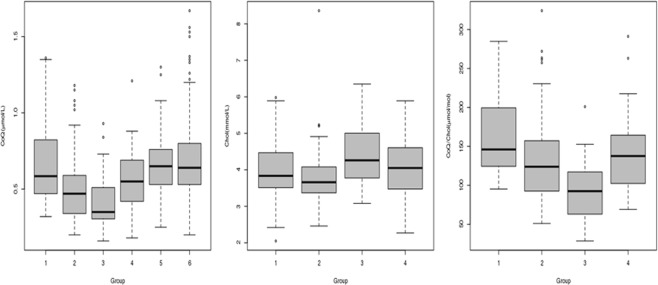
Box plot representation of: Left panel. Plasma CoQ values (µmol/L) in the 6 subject groups. *Middle panel*. Serum Chol (mmol/L) and Right panel CoQ/Chol values (µmol CoQ/mol Chol) from controls, PKU, MPS and IEM patients. Low plasma CoQ concentrations are frequently observed in both PKU and MPS patients, while the IEM group show intermediate values. The length of the boxes indicates the interquartile space (p25–p75); the horizontal line into the box represents the median (p50), and the circles indicate outlier values. *X-axis groups: Group 1: healthy participants. Group 2: phenylketonuric. Group 3: patients with mucopolysaccharidoses. Group 4: patients with other inborn errors of metabolism. Group 5: patients with neurogenetic diseases. Group 6: individuals with neurological diseases with no genetic diagnosis.

Plasma CoQ, Chol, CoQ/Chol ratio values, and the number of cases with low plasma CoQ values in the different cohorts of patients are stated in Table 1 and Fig. 2. ANOVA with Bonferroni correction showed that plasma CoQ values were significantly lower in the PKU and MPS groups than in controls and neurological patients. IEM group showed intermediate values that were not significantly different from those of the controls (Fig. 2, Tables 1 and 2). When we compared the CoQ/Chol ratios in these 3 groups, MPS values were significantly lower than controls, PKU, and IEM (Tables 1 and 2). In turn, IEM and PKU patients showed significantly lower values than controls (Table 2). The highest percentage of patients with low-plasma CoQ and Q/Chol values belonged to the MPS group. In this group, low plasma CoQ value was a consistent feature in Sanfilippo patients, but it was also present in other MPS patients, except for Hurler-Scheie and Maroteaux-Lamy patients (data not shown). Even MPS patients with normal CoQ values displayed plasma CoQ concentrations close to the lowest limit of our reference interval.

**Table 2:** ANOVA with Bonferroni correction showed that plasma CoQ values were significantly lower in the PKU and MPS groups than in controls.

Group (I)	Groups (J)	Average difference (I-J)	95% confidence interval
**CoQ**			
Controls	PKU	0.164	0.067–0.261 (p < 0.0001)
	MPS	0.252	0.127–0.377 (p < 0.0001)
	IEM	0.100	−0.013–0.213 (p = 0.139)
**CoQ/Chol**			
Controls	PKU	31.6	11.4–51.8 (p < 0.0001)
	MPS	69.4	38.9–99.9 (p < 0.0001)
	IEM	24.1	0.4–47.8 (p = 0.043)
MPS	PKU	−37.8	−67.4 – −8.4 (P = 0.003)
	IEM	−45.3	−77.3–13.4 (p = 0.001)

IEM group showed intermediate values that were not significantly different from those of the controls. When we compared the CoQ/Chol ratios in these 3 groups, MPS values were significantly lower than controls, PKU, and IEM. In turn, IEM and PKU patients showed significantly lower values than controls.

**Figure 3:**
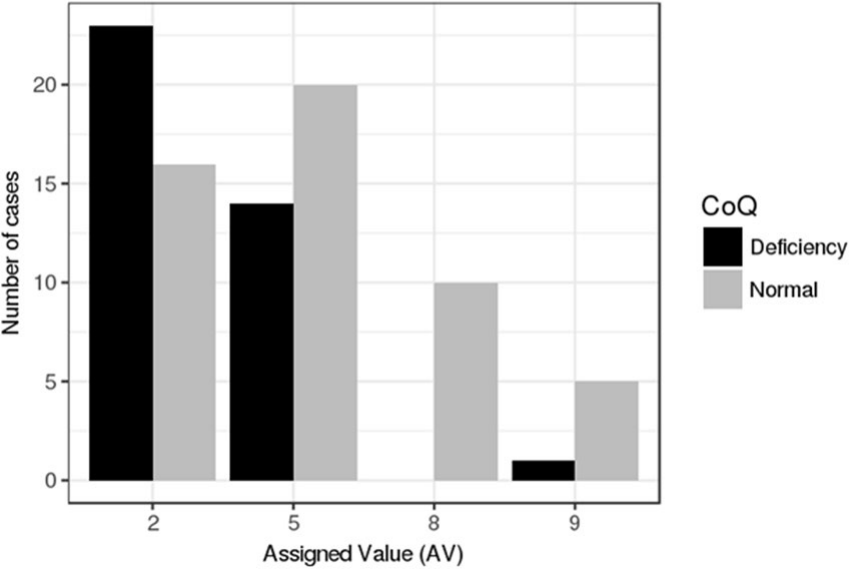
Graphic resprsentation of the assigned value (AV) study results in PKU patients. The black bars represent the number of PKU paints displaying low plasma CoQ concentrations in the different AV groups. The grey bars represent the number of PKU patients with normal plasma CoQ concentration. In AV groups 2 and 5 (classic PKU), the number of cases displaying low plasma CoQ values is higher than those belonging to AV groups 8 and 9 (milder PKU forms).

In 65 PKU cases, we studied the correlation between CoQ concentration, the residual predicted PAH activity, and the Phe value at diagnosis (as a predictor of disease severity). As expected, residual PAH activity was found to be negatively correlated with the Phe level at diagnosis (r = −0.448; p < 0.0001) but positively correlated with the CoQ concentration (r = 0.379; p = 0.003). To further validate this observation, we used the assigned value study by classifying 89 PKU patients into 4 groups (2, 5, 8, and 9) according to the type of mutation (Supplementary Data Set). Chi-Square test showed a significant association between having low plasma CoQ values and belonging to group 2 (Chi square = 7.518; p = 0.006). Thus, from the 39 cases of group 2, 23 displayed low CoQ values, while 16 were normal. In the 34 patients classified as group 5, 14 showed low CoQ values and 20 were normal. From the 16 cases belonging to groups 8 and 9, only 1 displayed low CoQ concentrations. Results are represented in Fig. 3.

Regarding the patients with IEM, the percentage of cases with low CoQ values was lower than those of MPS and PKU patients (Table 1), and the frequency of low CoQ values was randomly distributed in homocystinurias, organic acidurias, and urea cycle defects. We compared (student t test) the plasma CoQ and CoQ/Chol values between patients with IEM and carbohydrate-restricted diet (galactosemias and fructosemias) with those taking protein-restricted diets (amino acid defects and organic acidemias), and no differences were observed. Only plasma CoQ levels tended to be lower (p = 0.069) in the protein-restricted group, but values normalized when they were related to Chol.

The 2 groups of neurological patients did not show any significant differences in plasma CoQ concentrations compared with the control group values, and the percentage of cases with low CoQ values was below 8% (Table 1, Fig. 2). NGS analysis in selected neurological patients with low plasma CoQ values failed to detect any pathogenic mutations in genes related to CoQ biosynthesis or associated with secondary CoQ deficiency. However, we found two unexpected findings: one patient with an epileptic encephalopathy diagnosed with a channelopathy (CACNA1A (NM_023035.2): p.Arg198Gln/c.593 G > A (heterozygous) and another case diagnosed with a Xq28 duplication syndrome (chrX:154125883–154562336 duplication [436Kb] [heterozygous]). The pathogenicity of both mutations was assessed following the recommendations of the American College of Medical Genetics^25^.

## Discussion

To our knowledge, this is the first work to analyze plasma CoQ and Chol status in a large cohort of pediatric patients. We first confirmed the previously published reference intervals for CoQ and CoQ/Chol values in a healthy population of 83 individuals. The concentration of plasma CoQ in healthy subjects has been reported by different authors in various populations, in general with a good agreement and such values close to those stated in the present study^26–29^. Since CoQ is known to bind to lipoproteins, the amount of CoQ in the plasma can also be related to the amount of Chol, and differences in total CoQ may be normalized to total Chol^29^. Another important observation was that neither plasma CoQ nor CoQ/Chol ratios correlated with the age in the control group (all were below 22 years), establishing only one reference interval. These results are in agreement with other studies^29^ and indicate the stability of plasma CoQ status, at least in humans below 22 years.

Other remarkable observation of the present study is that low plasma CoQ values is a common biochemical feature in PKU and MPS patients and less frequent in the IEM group (in spite of having restricted diets that might lead to CoQ suboptimal status). In these groups, we also analyzed total Chol and calculated CoQ/Chol ratio to assess whether these values were concomitantly decreased. PKU is caused by a deficiency in the enzyme phenylalanine 4-hydroxylase (EC 1.14.16.1) due to mutations in the PAH gene. Plasma CoQ status is decreased in around one-third of the PKU patients compared to an age-matched reference population, as demonstrated previously^12^, as well as in the present study. Several hypotheses may explain this deficiency, although none of them have yet been demonstrated in humans, and a combination of different factors is the most plausible explanation: Firstly, PKU patients avoid foods that are rich sources of CoQ. Secondly, the availability of tyrosine is essential for the synthesis of CoQ. Tyrosine may be low in PKU patients, but no association has been reported between the lowered plasma tyrosine concentration of patients and their serum CoQ level^30^. Thirdly, other factor associated with the decreased plasma CoQ level of PKU patients may be the elevated blood phenylalanine concentrations^30^, since experimentally induced hyperphenylalaninemia in mice has been reported to inhibit the activities of the brain and liver enzymes, 3-hydroxy-3-methylglutaryl-CoA reductase, and mevalonate-5-pyrophosphate decarboxylase^31^, which are both essential for CoQ and Chol biosynthesis. Interestingly, plasma CoQ and Chol values were decreased in PKU patients, indicating that inhibition of the mevalonate pathway may be the common cause of the deficit in CoQ and Chol status. In all likelihood, the factors responsible for the CoQ deficiency in PKU are multifactorial, but the demonstration of an association between the predicted PAH residual activity or the type of *PAH* mutations and the CoQ status of the patients supports the hypothesis that the more severe the degree of hyperphenylalaninemia, the lower the level of plasma CoQ. Therefore, the inhibition of the mevalonate pathway by high Phe values^30,31^ would be the most plausible explanation for decreased concentrations of both Chol and CoQ.

Regarding the MPS group, only 2 reports demonstrate that a noticeable percentage of patients with a genetic diagnosis of MPS present with plasma CoQ deficiency^15,32^. Those studies did not conduct etiologic investigations but raised several hypotheses regarding the presence of low plasdma CoQ values: (1) Impaired liver function in MPS, which can cause low plasma CoQ levels; (2) Nutritional problems, although this was unlikely since other lipophilic and hydrophilic vitamins were normal in these patients^15,32^; (3) the most plausible explanation for the deficit in plasma CoQ concentrations is related to vitamin B_6_, which can show low blood levels in MPS patients^32^. The active form of vitamin B_6_, pyridoxal 5-phosphate, is required for the transamination of tyrosine into 4-hydroxyphenylpyruvic acid for CoQ biosynthesis. In this context, a correlation between blood CoQ and vitamin B_6_ status has been demonstrated^33^. It is also unknown whether CoQ deficiency is also present in tissues and how this may contribute to the pathophysiology of MPS. In the present study, this group displayed the highest frequency and most profound low plasma CoQ concentrations, while, in contrast to the PKU group, Chol values were not reduced in parallel. Furthermore, the low CoQ values was present in all types of MPS patients except for Hurler-Scheie and Maroteaux-Lamy patients. A hypothesis that may account for the CoQ subpotimal status in MPS is that heparan sulphate (and probably other mucopolysaccharides), may create adducts with pyridoxal 5-phosphate, leading to a loss of vitamin B_6_ and consequently low CoQ concentrations^32^. However, this has yet to be confirmed or refuted and requires further investigation.

Few reports have stated the association between other IEM under restricted dietary treatments and CoQ status. CoQ deficiency has been reported to be associated with propionic and type II glutaric acidurias^14,34,35^, mainly in muscle biopsy. We demonstrate here that none of the groups investigated showed a more consistent CoQ deficiency compared with the others. In fact, the IEM group displayed intermediate values between those for MPS, PKU, and the controls or the neurological patients. A limitation of the present study is that the IEM group is heterogeneous, and one hypothesis that can be drawn is that the contribution of the restricted diets to the plasma CoQ concentration would be expected to be limited (lack of differences between the CoQ values in carbohydrate-restricted diet group with that of the normal CoQ availability vs protein-restricted diet group that would be at risk of low CoQ intake). Thus, the contribution of dietary sources, which has been reported to be up to 25% of the total plasma CoQ values^3^, in the absence of other etiologic mechanisms such as in PKU, would explain these intermediate values. Interestingly, the degree of low CoQ values in this group was higher than that in the neurological patients, advocating the value of plasma CoQ monitoring in IEM patients, considering that these patients will be chronically treated.

Surprisingly, neither the neurogenetic group (including, for example, mitochondrial disorders patients) nor the non-diagnosed neurological patients showed reduced plasma CoQ values. Furthermore, the average plasma CoQ values in these 2 groups of patients were almost identical to those of the control group. Moreover, the NGS assessment of those selected cases with a complex neurological phenotype and CoQ deficiency failed to detect pathogenic mutations in the candidate genes, confirming that plasma CoQ status is quite stable, probably tightly regulated, and may not be a good biomarker to reflect systemic (brain, muscle) CoQ status. Thus, plasma CoQ would not be a good biomarker to demonstrate genetic diseases associated with CoQ deficiency. In fact, a high percentage of mitochondrial disease patients and those having primary genetic disturbances in CoQ biosynthesis show a CoQ deficient status in tissues (fibroblast, muscle) but not in plasma^36^. However, it has been demonstrated after a genome-wide association study that serum CoQ levels identify susceptibility loci linked to neuronal disease (Alzheimer’s disease, autism, and schizophrenia)^37^. No patients with these diseases were investigated in the present study, and this would explain these differences.

CoQ values may be influenced by several genetic and environmental conditions, including changes in dietary habits and cholesterol patterns. Since, therefore the variation amongst individuals may be great, single and isolated CoQ determinations in patients are probably no sufficient for detecting a real CoQ suboptimal status or for indicating CoQ supplementation.

In conclusion, low plasma CoQ values is neither a common finding in most of neuropediatric patients nor a good biomarker to predict genetic conditions leading to primary CoQ deficiency. On the contrary, plasma CoQ monitoring in selected groups of patients with different IEM (especially in PKU and MPS patients, but also in organic acidemias and aminoacidopathies under protein-restricted diets) seems advisable to prevent the possibility of a chronic low blood CoQ values in such groups of patients.

## Supplementary information

Supplementary material 1 and figure 1.

Dataset 1

## Data Availability

The datasets generated during and/or analysed during the current study are available from the corresponding author on reasonable request.
