# Supplementary material for: Plasma coenzyme Q10 status is impaired in selected genetic conditions
Source: Sci Rep. 2019 Jan 28;9:793. doi: 10.1038/s41598-018-37542-2 (PMC6349877; doi:10.1038/s41598-018-37542-2)

Plasma coenzyme Q_{10} status is impaired in selected genetic conditions.

Running title: Coenzyme Q_{10} and genetic diseases.

Raquel Montero [1,2], Delia Yubero [1], Maria C. Salgado [1], María Julieta González [1], Jaume Campistol[1,2], Maria del Mar O`Callaghan [1,2], Mercè Pineda[1], Verónica Delgadillo[1], Joan Maynou[3], Guerau Fernandez [3], Julio Montoya [2,4], Eduardo Ruiz -Pesini[2,4], Silvia Meavilla[1], Viruna Neergheen [5], Angels García -Cazorla[1,2], Placido Navas [2,6], Iain Hargreaves[5,7], Rafael [Artuch*1,2].

[1]Inborn errors of metabolism Unit. Institut de Recerca Sant Joan de Déu. Barcelona, Spain.

[2]CIBER de Enfermedades Raras (CIBERER), Instituto de Salud Carlos III Spain.

[3]Department of Genetic and Molecular Medicine, Institut de Recerca Sant Joan de Déu. Barcelona, Spain.

[4]Departamento de Bioquimica y Biologia Molecular y Celular, Universidad Zaragoza - Instituto de Investigación Sanitaria de Aragón (IISAragon), Zaragoza, Spain

[5] Neurometabolic Unit, National Hospital, Queen Square, London, UK.

[6]Centro Andaluz de Biología del Desarrollo. Universidad Pablo de Olavide, Sevilla, Spain.

[7]School of Pharmacy, Liverpool John Moores University, Liverpool, UK

***Corresponding author**: rartuch@hsjdbcn.org

Supplementary material 1. Patient groups description.

PKU (n=113): table with patient mutations and the assigned value, when available (n=89), are reported in supplementary table 1.

MPS (n=44): MPS I Hurler- Scheie (4), MPS I Hurler (2), MPS II Hunter (2), MPS III (Sanfilippo) A (16), MPS IIIB (5), MPS IIIC (7), MPS IV Morquio (4), MPS VI Maroteaux-Lamy (3) and MPS VII Sly (1).

IEM (n=61): 11 organic acidurias (glutaryl CoA dehydrogenase, propionyl -CoA carboxylase and 3 -hydroxy-3-methylglutaryl-Coa lyase deficiencies): 20 urea cycle disorders (ornithine carbamoyltransferase deficiency, carbamoyl phosphate synthetase I deficiency, lysinuric with protein intolerance, hyperornithinemia -hyperammonemia-homocitrullinuria, citrullinemia and argininosuccinic aciduria), 12 homocystinurias (cystathionine beta -synthase, methylmalonic aciduria and homocystinuria cblC type, homocystinuria-megaloblastic anemia cblE type, 10 classic galactosemias, 2 fructose intolerance and 6 aminoacidopathies (tysorinemia type i, maple syrup urine disease and nonketotic hyperglycinemia).

Neurogenetic conditions (n=99): 34 Friedreich ataxia, 18 mitochondrial diseases (9 mtDNA mutations; MELAS, NARP, Pearson and MERFF syndromes) and 9 in nuclear genes (*TSFM, TUFM, POLG, SPG7, TIMM8A, BCSL1 and FRAS2*), 20 neuromuscular diseases (11 type II and 6 type III spinal amyotrophy and 3 colagenopathies) and 27 miscellaneous (GLUT -1 deficiency, CDG syndromes, complex molecule disorders (lysosomal and peroxisomal disorders), Rett syndrome, channelopaties and other neurogenetic syndromes.

Neurological and other conditions with no diagnosis (n=197): neuropediatric patients with no genetic diagnosis with 3 main categories: A) predominant neurological

phenotype (n=140) 1) Epilepsy and epileptic encephalopathies; 2) movement disorders (mainly dystonia and ataxia). 3) other (hypotonia and mental dissability). B) Neuromuscular disorders (n= 10). C) Neurological phenotype plus other organ involvement (endocrine, neurosensorial, nephrological and cardiological) (n=47).

Supplementary data set: We applied the a ssigned value study to classify 89 PKU patients into 4 groups (2, 5, 8, and 9) according to the type of mutation in each allele. The sum of the scores obtained for every allele led to the final classification of patients (right column; total AV value).

<u>Supplementary Figure 1 :</u> Scatterplot representation of the correlation between plasma CoQ and total cholesterol values in the control population.

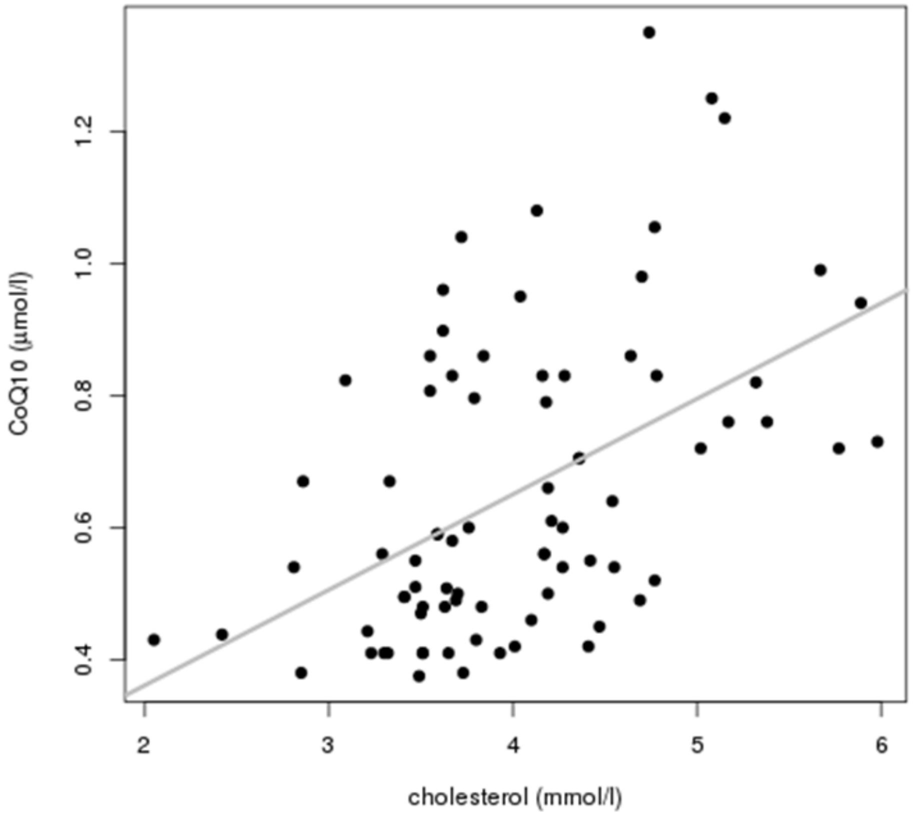

Supplement: Supplementary file 1 — Supplementary material 1 and figure 1. [file 41598_2018_37542_MOESM1_ESM.pdf]
